# Non-therapeutic male circumcision in infancy or childhood and risk of human immunodeficiency virus and other sexually transmitted infections: national cohort study in Denmark

**DOI:** 10.1007/s10654-021-00809-6

**Published:** 2021-09-26

**Authors:** Morten Frisch, Jacob Simonsen

**Affiliations:** 1 Department of Epidemiology Research, Statens Serum Institut, 2300 Copenhagen S, Denmark; 2 Department of Clinical Medicine, Center for Sexology Research, Aalborg University, 9000 Aalborg, Denmark

**Keywords:** Circumcision, Cohort study, Human immunodeficiency virus, Anogenital warts, Syphilis, Sexually transmitted infections

## Abstract

Whether male circumcision in infancy or childhood provides protection against the acquisition of human immunodeficiency virus (HIV) or other sexually transmitted infections (STIs) in adulthood remains to be established. In the first national cohort study to address this issue, we identified 810,719 non-Muslim males born in Denmark between 1977 and 2003 and followed them over the age span 0–36 years between 1977 and 2013. We obtained information about cohort members’ non-therapeutic circumcisions, HIV diagnoses and other STI outcomes from national health registers and used Cox proportional hazards regression analyses to calculate hazard ratios (HRs) with 95% confidence intervals (CIs) associated with foreskin status (i.e., circumcised *v.* genitally intact). During a mean of 22 years of follow-up, amounting to a total observation period of 17.7 million person-years, 3375 cohort members (0.42%) underwent non-therapeutic circumcision, and 8531 (1.05%) received hospital care for HIV or other STIs. Compared with genitally intact males, rates among circumcised males were not statistically significantly reduced for any specific STI. Indeed, circumcised males had a 53% higher rate of STIs overall (HR = 1.53, 95% CI: 1.24–1.89), and rates were statistically significantly increased for anogenital warts (74 cases in circumcised males *v*. 7151 cases in intact males, HR = 1.51; 95% CI: 1.20–1.90) and syphilis (four cases in circumcised males *v*. 197 cases in intact males, HR = 3.32; 95% CI: 1.23–8.95). In this national cohort study spanning more than three decades of observation, non-therapeutic circumcision in infancy or childhood did not appear to provide protection against HIV or other STIs in males up to the age of 36 years. Rather, non-therapeutic circumcision was associated with higher STI rates overall, particularly for anogenital warts and syphilis.

## Introduction

The World Health Organization (WHO) has endorsed voluntary medical male circumcision (VMMC) for adult men and boys above 15 years of age as an “*efficacious HIV prevention option (…) in settings with generalized epidemics to reduce the risk of heterosexually acquired HIV infection*” [1]. This policy rests on the finding of overall 58% *efficacy* against female-to-male HIV transmission in three randomized controlled trials (RCTs) in Kenya, Uganda and South Africa conducted in the early 2000s [2–5]. These trials also suggested a lowered risk of various STIs following VMMC [6–8]. A prospective study from the research group behind one of the RCTs [4] recently confirmed their original findings in a study covering 30 agrarian and semi-urban trading communities in Uganda [9]. While this new finding has been interpreted as proof of VMMC’s real-world effectiveness against HIV acquisition [10], the actual implications are inconclusive. As acknowledged by the authors, the new Uganda study failed to adjust for the impact of pre-exposure antiretroviral prophylaxis (PrEP), which is arguably the single most important biomedical determinant of HIV acquisition [11–13], and this omission may well have skewed the reported association of VMMC with HIV risk. Moreover, as also noted by the authors [9], observed major demographic and behavioral differences between genitally intact and circumcised men in the study left ample room for residual confounding.

VMMC programs in eastern and southern parts of Africa have so far resulted in well over 23 million circumcisions since 2004 [1, 14]. However, a considerable proportion of men in target countries remain reluctant to undergo such surgery. Questions surrounding VMMC’s real-world effectiveness, combined with increasing knowledge about more effective and less invasive means of HIV prevention, reports of negative sexual consequences among some men undergoing the procedure at various ages [15–19], and bioethical concerns [20] may be among the relevant factors in explaining such reluctance.

Given that men in many target regions are not volunteering for circumcision at the rates set by official quotas [21], attempts have been made to increase parental acceptability of early infant male circumcision in high-risk settings [22–24], so far with limited success [25]. However, no RCT or carefully conducted observational study with long-term follow-up has ever demonstrated an HIV-protective effect of circumcision performed in infancy or childhood. Moreover, as of 2020, concerns about markedly increased rates of serious glans injuries, urethrocutaneous fistulas and other surgical complications following circumcision in infancy or childhood compared to late adolescence or adulthood prompted the U.S. President’s Emergency Plan For Aids Relief (PEPFAR) to halt financial support for circumcisions prior to Tanner stage 3 development [14, 26].

To address the paucity of relevant evidence in this area, we here present the results of the first-ever nationwide cohort study of whether non-therapeutic male circumcision in infancy or childhood provides long-term protection against the acquisition of HIV and other STIs in adolescence and adulthood. As in the recent study from Uganda [9], we restricted the focus to individuals with a non-Muslim family background to reduce the potential confounding influence of cultural or behavioral risk factors that may differ between Muslims and non-Muslims. Consequently, we provide estimates of the relative risk of acquiring HIV and other STIs up to more than three decades after infant or childhood circumcision, comparing hazard rates for such infections among circumcised and genitally intact non-Muslim males in Denmark.

## Methods

### Cohort

We used the Civil Registration System to identify 855,654 males born in Denmark (excluding Greenland and the Faroe Islands) between January 1, 1977 and December 31, 2003 [27]. By means of a demographic algorithm that we used in two previous studies on possible outcomes of non-therapeutic circumcision [28, 29], we excluded 44,935 males with at least one parent or grandparent born in one of the following 17 predominantly Muslim countries: Turkey, Iraq, Pakistan, Iran, Somalia, Lebanon, Afghanistan, Morocco, Egypt, Syria, Indonesia, Algeria, Jordan, Bangladesh, Kuwait, Tunisia and Kosovo. No other predominantly Muslim country accounts for more than 0.1% of all non-Danish born citizens. The remaining 810,719 males constituted our study cohort of Danish-born non-Muslim males, whom we followed for the occurrence of HIV infection and other STIs over the age span 0–36 years between January 1, 1977 and November 30, 2013.

### Exposure categories

Cohort members who underwent non-therapeutic circumcision in a hospital setting were identified in the National Patient Register under surgery codes 55620 (period 1977–1995) or KKGV20 (since 1996) [30]. Non-therapeutic circumcisions performed in private clinics by surgeons or gynecologists and subsidized by the national healthcare system were identified under disbursement code 5301 in the National Health Service Register (since 1994) [31]. From 2004 onwards, non-therapeutic circumcision was gradually removed from regional lists of publicly subsidized surgical procedures, thus resulting in incomplete records for non-therapeutic circumcisions performed after 2003.

We also searched the files of these registries for all recorded cases of foreskin surgery other than non-therapeutic circumcision (e.g., phimosis surgery), using National Patient Register surgery codes 56640, 56680, 56700, 56720, or 56760 (period 1977–1995) and KKGH10, KKGV10, KKGV00, KKGH80, or KKGH80A (since 1996), and disbursement codes 3101, 3132, 3201, 3232, 4132, 4154, 4232, or 6422 in the National Health Service Register (since 1994) [29]. We used this information to enable clean comparisons of rates of HIV infection and other STIs in males undergoing non-therapeutic circumcision *v* intact males by censoring cohort members on the date of such other foreskin surgery, as explained below.

### Outcomes

Specific diagnostic codes according to the International Classification of Diseases, 8^th^ (ICD-8, 1977–1993) and 10th edition (ICD-10, 1994–2013) were used to identify cohort members treated in Danish hospitals for HIV (ICD-8: 07983; ICD-10: B20-B24), syphilis (ICD-8: 091xx-097xx; ICD-10: A51-A53), gonorrhea (ICD-8: 098xx; ICD-10: A54), genital herpes (ICD-8: 05402; ICD-10: A60), anogenital warts (ICD-8: 09990; ICD-10: A630-A630D), or other STIs, including chlamydia, chancroid, granuloma inguinale and trichomoniasis (ICD-8: 07984, 099xx (except 09990), 131xx; ICD-10: A55-A59, A63-A64 (except A630-A630D)) between January 1, 1977 and November 30, 2013.

### Analysis

In separate analyses for each STI, we followed cohort members from their date of birth until the date of first recorded STI diagnosis, emigration, death or end of follow-up on November 30, 2013, which was the date of complete registry records at the time of data extraction for this and a previous study [29], whichever came first. All statistical analyses of the association of foreskin status with risk of HIV infection and other STIs during 1977–2013 were carried out as Cox proportional hazards regression analyses with age as the underlying time scale, stratifying the baseline hazard rates for birth year [32]. Specifically, hazard ratios (HRs) with 95% confidence intervals (CIs) compared the hazard rates of each outcome between the reference group of intact males and males undergoing non-therapeutic circumcision. Each cohort member’s foreskin status was treated as a time-dependent variable being intact from birth and, when relevant, shifting to circumcised on the recorded date of non-therapeutic circumcision. Boys and men undergoing other foreskin surgery as described above were censored on the date of such surgery.

In a subsequent analysis, we examined the extent to which observed associations for any STI and for anogenital warts depended on the age when the non-therapeutic circumcision took place. Specifically, we calculated HRs comparing rates of any STI and of anogenital warts among males who underwent non-therapeutic circumcision at or before the age of 24 months and those who had this surgery after that age with corresponding rates in the reference category of intact males.

To compare observed rates of HIV acquisition among intact and circumcised males, we undertook a simulation study. Specifically, we conducted a series of 100,000 simulations to establish the probability distribution of anticipated numbers of cases of HIV infection among circumcised males in the cohort assuming no association between foreskin status and HIV risk. Such an approach was appropriate because the average periods of follow-up were almost identical in the compared groups, being 21.8 years among intact males and 21.6 years among circumcised males. Among international organizations and stakeholders arguing in favor of circumcision in the fight against HIV in Africa, the expectation is that infant or childhood circumcision will provide the same, or an even higher, degree of HIV protection compared to what has been reported for adult male circumcision [14, 22–25]. Consequently, we tested the directional (one-sided) null hypothesis of equal or higher rates of HIV acquisition in circumcised males against the alternative hypothesis that the rate is lower among circumcised males.

### Robustness analyses

In one robustness analysis aimed to address the potential impact of socioeconomic confounding, we repeated the main analysis outlined above, this time stratifying for a variable capturing municipality-based average disposable household income level in quintiles (273,760–305,458, 305,742–322,403, 323,556–339,384, 340,184–358,568 and 359,050–599,126 Danish Kroner) (Statistics Denmark, www.statistikbanken.dk). Specifically, we treated this municipality-based socioeconomic variable as a time-dependent stratification variable, whose value depended on each cohort member’s actual day-to-day place of residence during follow-up.

In a second robustness analysis, we ended follow-up for HIV and other STIs on December 31, 2003 to eliminate any exposure misclassification due to incomplete data on non-therapeutic circumcisions performed after 2003.

By restricting our cohort to non-Muslims, using birth year as a stratification variable for the baseline hazard rates, using age as the underlying time scale in all analyses, and censoring on the date of other foreskin surgery, we ensured that all HRs were based on culturally comparable, same-aged strata of circumcised and intact males observed during comparable calendar years.

All Cox proportional hazards regression analyses were carried out using the PHREG procedure in SAS, version 9.4 (SAS Institute, Cary, NC, USA).

## Results

In our cohort of 810,719 non-Muslim males, the total observation period at risk for any STI between January 1, 1977 and November 30, 2013 was 17.6 million person-years in intact males and 73,032 person-years in circumcised males. During follow-up, 3375 males (0.42%) were recorded as having undergone non-therapeutic circumcision in a hospital department or a doctor’s clinic. For cohort members born 1994 to 2003, when circumcision data were available from both hospital departments and private clinics, the median age at circumcision was 5.9 months (range 0 days–10 years, interquartile range 2.4 years).

### Non-therapeutic circumcision and risk of any STI

Overall, 8531 cohort members were diagnosed in a Danish hospital with any STI, with the rate being 53% higher among circumcised than intact males (HR = 1.53; 95% CI: 1.24–1.89) (Table 1).

**Table 1:** Hazard ratios (95% confidence intervals) of hospital contacts for sexually transmitted infections according to foreskin status among 0–36 year-old non-Muslim males, Denmark 1977–2013

	Foreskin status	Cases	Person-years	HR^a^	(95% CI)	HR^b^	(95% CI)
Any STI	Intact	8,444	17,625,247	1	Ref	1	Ref
	Circumcised	87	73,032	1.53	(1.24–﻿1.89)	1.44	(1.17–﻿1.78)
Anogenital warts	Intact	7,151	17,632,874	1	Ref	1	Ref
	Circumcised	74	73,100	1.51	(1.20–1.90)	1.43	(1.14–1.79)
Gonorrhea	Intact	364	17,667,813	1	Ref	1	Ref
	Circumcised	5	73,558	2.30	(0.95–5.57)	2.23	(0.92–5.38)
HIV/AIDS	Intact	321	17,667,559	1	Ref	1	Ref
	Circumcised	0	73,576	–	NS	-	NS
Syphilis	Intact	197	17,668,780	1	Ref	1	Ref
	Circumcised	4	73,562	3.32	(1.23–8.95)	3.25	(1.21–8.78)
Genital herpes	Intact	197	17,668,242	1	Ref	1	Ref
	Circumcised	2	73,573	1.54	(0.38﻿–6.19)	1.50	﻿(0.37–6.06)
Other STIs^c^	Intact	609	17,665,434	1	Ref	1	Ref
	Circumcised	3	73,540	0.75	(0.24﻿–﻿2.34)	0.74	(0.24–2.29)

*HR* hazard ratio, *CI* confidence interval, *NS* not statistically significant, *STI* sexually transmitted infection

Cohort of 810,719 Danish-born non-Muslim males born January 1977 through December 2003 and followed for sexually transmitted infections between January 1977 and November 2013

^a^Hazard ratios stratified for birth year with age as the underlying time scale

^b^Hazard ratios stratified for birth year and municipality-based household income with age as the underlying time scale

^c^Other STIs include chlamydia, chancroid, granuloma inguinale and trichomoniasis

### Non-therapeutic circumcision and risk of specific STIs other than HIV

Anogenital warts, affecting 7225 males (74 circumcised, 7151 intact), were by far the most common recorded type of STI in the cohort, with the rate being statistically significantly higher in circumcised than intact males (HR = 1.51; 95% CI: 1.20–1.90). The rate of gonorrhea in circumcised males was also higher than in intact males (HR = 2.30; 95% CI: 0.95–5.57), though not statistically significantly so (5 circumcised, 364 intact). Despite small numbers among circumcised males (4 circumcised, 197 intact), the rate of syphilis was more than threefold higher in circumcised males (HR = 3.32; 95% CI: 1.23–8.95). Findings for genital herpes and the composite group of other STIs were inconspicuous (Table 1).

We examined whether the HRs for any STI and for anogenital warts specifically differed according to the age at which the non-therapeutic circumcision took place. Compared with intact males, HRs for any STI were similar for males undergoing circumcision at or before (HR = 1.57; 95% CI: 0.99–2.49) or after (HR = 1.52; 95% CI: 1.20–1.92) the age of 24 months, based on 18 STIs in males circumcised at or before the age of 24 months, 69 STIs in males circumcised after that age, and 8444 STIs in intact males. Similarly, compared with intact males, HRs for anogenital warts were elevated for males undergoing non-therapeutic circumcision both at or before (HR = 1.73; 95% CI: 1.07–2.78) and after (HR = 1.46; 95% CI: 1.13–1.90) the age of 24 months, based on 17 cases of anogenital warts in males circumcised at or before the age of 24 months, 57 cases in males circumcised after that age, and 7151 cases in intact males.

### Non-therapeutic circumcision and risk of HIV

A total of 321 cohort members acquired HIV infection during follow-up (0 circumcised, 321 intact). To meaningfully compare these numbers, the observation time at risk in the two groups must be taken into account. Among circumcised males, the 73,576 person-years at risk constituted only 0.4% of the total observation time, so to evaluate if the zero cases of HIV infection in this group differed significantly from the rate of 321 cases during 17.7 million person-years in intact males, we performed a simulation study (Table 2). Specifically, in a series of 100,000 simulations we established the probability distribution of anticipated numbers of cases of HIV infection among circumcised males assuming similar rates of HIV in the two groups, a plausible method considering the almost identical average periods of follow-up among intact (21.8 years) and circumcised (21.6 years) cohort members. As seen in Table 2, under the directional null hypothesis of equal or higher rates of HIV acquisition in circumcised males, zero observed cases of HIV infection among circumcised males occurred in 9.4% of the simulations. In other words, with a one-sided p value of 0.094 the null hypothesis was not rejected, implying that rates of HIV infection among circumcised and intact males were not statistically significantly different.

**Table 2:** Probability distribution for number of cases of HIV infection occurring in circumcised males assuming similar underlying rates of HIV acquisition among circumcised and intact 0–36 year-old non-Muslim males, Denmark 1977–2013

Number of cases of HIV infection in circumcised males	Frequency (in 100,000 simulations)	Probability (%)
0	9349	9.35
1	22,224	22.22
2	25,805	25.81
3	20,858	20.86
4	12,372	12.37
5	5893	5.89
6	2400	2.40
7	787	0.79
8	224	0.22
9	61	0.06
10	23	0.02
11	3	0.003
12	1	0.001

### Robustness analyses

In the first robustness analysis, we aimed to take the possible confounding influence of socioeconomic factors into account by stratifying for municipality-based disposable household income. This analysis yielded similar HRs as in the main analysis, including for any STI (HR = 1.44; 95% CI: 1.17–1.78), anogenital warts (HR = 1.43; 95% CI: 1.14–1.79) and syphilis (HR = 3.25; 95% CI: 1.21–8.78) (Table 1).

In the second robustness analysis, we ended follow-up on December 31, 2003 to reduce exposure misclassification. Due to the maximum attained age of 26 years during follow-up in this robustness analysis, the overall observed number of STIs (n = 1148 cases) was markedly reduced compared with the main analysis, thus rendering most analyses for specific STIs statistically unstable. However, the overall HR for any STI was similar to that of the main analysis (HR = 1.54; 95% CI: 0.89–2.65, based on 13 cases in circumcised males *v* 1135 cases in intact males), whereas the association of circumcision with risk of anogenital warts was strengthened (HR = 1.79; 95% CI: 1.01–3.17, based on 12 cases in circumcised males *v* 869 cases in intact males).

## Discussion

To the best of our knowledge, our study is the first to offer a nationwide, prospective assessment of the association between circumcision in infancy or childhood and subsequent risk of acquiring HIV and other STIs. Until now, only two small longitudinal studies from New Zealand have addressed this question [33, 34]. In a 1977 birth cohort from Christchurch followed to the age of 25 years, researchers combined early maternal reports about circumcision status and self-reported STI histories at age 21 and 25 years for their assessment. Overall, 154 participants (30%) were categorized as circumcised before age 15 years, and 44 (9%) reported at least one episode of STI. Logistic regression analyses yielded statistically non-significant associations of circumcision with overall STI risk in cohort-specific follow-up windows in age groups 18–21 years and 21–25 years as well as for individual STIs studied, including chlamydia, the most common STI reported by 22 cohort members. When considering all STIs together and combining data from the two age groups, the authors noticed a statistically significant association with higher odds of any STI among intact than circumcised participants (odds ratio = 2.7; 95% CI: 1.2–6.1) [33].

In another birth cohort comprising 499 men born 1972–1973 in Dunedin, participants were followed for STI acquisition to age 32 years. According to maternal reports, 201 cohort members (40%) had been circumcised before age 3 years, and 117 (23%) reported having had one or more STIs, notably genital warts (56 cases), chlamydia (46 cases) and genital herpes (34 cases). With almost three times more STI cases during follow-up than in the Christchurch study, the Dunedin cohort had substantially greater statistical power to identify a potential protective effect of circumcision in infancy or childhood against STI acquisition, should one such truly exist. However, when comparing intact males with the reference group of circumcised males, the Dunedin study revealed no indication of a protective effect of circumcision against STI acquisition overall (incidence rate ratio (IRR) = 1.0; 95%CI: 0.7–1.3), against bacterial STIs combined (IRR = 1.1; 95%CI: 0.6–2.0), against viral STIs combined (IRR = 0.9; 95%CI: 0.6–1.4) or against any of the individual bacterial or viral STIs studied [34].

While numbers of circumcised males who acquired an STI were altogether seven in the Christchurch study [33] and 47 in the Dunedin study [34] during follow-up of cohort members until age 25 and 32 years, respectively, our study identified 87 STI cases in circumcised males followed for up to 36 years. For intact males, numbers acquiring an STI were 37 in the Christchurch study, 70 in the Dunedin study and 8444 in our study. Thus, our nationwide cohort study in Denmark is by far the most powerful prospective study to date.

In contrast to findings from African RCTs of circumcisions carried out in adolescence and adulthood, which together suggested 58% efficacy of VMMC in providing short and intermediate term prevention of HIV [2–5], our national cohort study with up to more than three decades of follow-up found no reduction in risk of HIV or other STIs following circumcision in infancy or childhood. Consequently, our findings question the relevance and justifiability of ongoing efforts to expand VMMC programs in Africa to also cover circumcision of male infants and boys under 15 years of age [22–25]. If childhood circumcision provides no protection against the acquisition of HIV and other STIs in adulthood, as our findings and those of the Dunedin cohort study suggest [34], this knowledge should inform debates over the practice of non-therapeutic circumcision of male minors [35–39].

In a comprehensive meta-analysis of studies on the association of circumcision status with subsequent incidence and prevalence of STIs other than HIV, Van Howe reported slightly, yet non-significantly lower incidence rates of any STI (relative risk = 0.91; 95% CI: 0.78–1.07) and of syphilis (relative risk = 0.93; 95% CI: 0.64–1.34) among intact compared with circumcised men [40]. For several specific STIs, patterns were unclear, showing substantial differences between analyses based on STI incidence and prevalence data. For instance, the prevalence odds of genital warts were significantly lower in intact than circumcised males (odds ratio = 0.78; 95% CI: 0.63–0.96), but no significant difference was seen in prospective studies after attempts to correct HPV incidence rate ratios for uneven observation periods (lead time bias) and differential HPV detection probabilities among intact and circumcised men (relative risk = 0.96; 95% CI: 0.85–1.09). Overall, with due reservations, Van Howe concluded that while most specific STIs appear not to be impacted by circumcision status, the relative risks of any STI and, specifically, of genital warts appear to be lower in intact males [40]. With the numerically unstable exception of syphilis, which was significantly elevated among circumcised males in the present Danish study, our main findings of increased incidence rates of any STI and of anogenital warts in circumcised males are consistent with the conclusions reached by Van Howe.

## Strengths and limitations

The findings of our study should be viewed in light of its strengths and limitations. Foremost among its assets is that we used truly population-based data on both exposure (foreskin status) and outcomes (STIs) that were routinely collected in national registers for purposes unrelated to our current research question. Consequently, unlike studies based on ad hoc collected research data under study-specific circumstances, whose findings may be hard to generalize to the entire population, we relied on national register data in a country recognized for its favorable epidemiological research opportunities due to its tradition over many decades for keeping detailed registers of health-related matters covering the entire population [41].

We constructed our cohort using high-quality administrative data on hospital contacts and publicly paid subsidies to private practitioners [30, 31] to identify cohort members who had undergone non-therapeutic circumcision. We identified study outcomes in the National Patient Register [30], and we used information about other foreskin operations to enable a clean comparison of STI risks in circumcised and genitally intact males. Due to the free and easy access to health care for all citizens in Denmark, we consider it unlikely that the probability of having an established STI diagnosis recorded in the National Patient Register would differ between intact and circumcised males. Indeed, we believe our register data served as a valid population-based resource enabling an unbiased assessment of the relative risk of STIs in circumcised *v* intact males.

Another strength is that we restricted our study population to Danish citizens without a Muslim cultural background, using an algorithm that was previously found to be valid [28, 29]. A recent national sex survey showed that Danish Muslims differ from the non-Muslim majority population on several accounts, including on matters like partner status, perceived quality of partner relation, sexual identity, age at sexual debut, recent and lifetime number of sex partners, unsafe sexual practices, and payment for sex, all of which may impact the risk of acquiring HIV and other STIs [42]. The restriction of our cohort to non-Muslim males minimized the risk for potential confounding of the underlying true association of early-life male circumcision with risk of HIV and other STIs from unmeasurable factors that may differ among ethnic groups with different sexual attitudes and practices.

A number of limitations need consideration. First of all, our study was carried out in a country where non-therapeutic circumcision is an uncommon procedure. Specifically, only 0.42% of our non-Muslim cohort members were recorded as being circumcised for non-therapeutic reasons, so numbers of STIs in circumcised males were limited for all specific types of STI, except anogenital warts. Despite this limitation, our study had sufficient statistical power to show with 95% confidence that the true circumcision-associated HRs for any STI, anogenital warts and syphilis are at least 24%, 20% and 23% elevated, respectively. Moreover, in a directional (one-sided) test favoring circumcised genitalia, a test situation dictated by the prevailing expectation among organizations and stakeholders promoting circumcision in Africa that rates of HIV will be lower among circumcised males [14, 22–25], we failed to identify a difference in HIV risk between intact and circumcised males.

The infectious outcomes in our study comprised only those STIs among cohort members that were diagnosed in hospital departments, including ambulatory contacts in STI clinics. We had no means to identify STIs treated by general practitioners, which likely explains the relatively low numbers of common STIs such as chlamydia and genital herpes in our study. It is important to note, however, that while *absolute risk* estimates for the studied STIs among intact and circumcised males cannot be derived from our study, it most likely provides unbiased estimates of the underlying true *relative risks* of acquiring HIV and other STIs. This will be so, provided the ascertainment of such infections took place independently of the foreskin status of cohort members. To this end, in a culturally homogeneous cohort like the present it seems implausible that any man’s decision to contact the health care system with signs or symptoms of an STI, and any doctor’s ability to diagnose STIs in these men, would somehow systematically depend on the foreskin status of these patients. Consequently, we consider our *relative risk* estimates, including the statistically significantly increased HRs for any STIs, anogenital warts and syphilis, as being reliable, regardless of our inability to provide accurate measures of *absolute* *risk*.

Relying on register data, our study did not have information available about the sexual practices of cohort members. Consequently, we cannot tell if, and to what extent, different sexual behaviors may have contributed to the observed higher STI risk in circumcised males. Links between childhood circumcision status and sexual behaviors later in life are not well-established, but some notable findings are worthy of consideration. In a study from the United States, where most non-therapeutic circumcisions are carried out shortly after birth, circumcised men tended to engage in a somewhat more elaborated set of sexual practices than intact men [43], which is consistent with a recent study showing higher sociosexual activity, including higher partner numbers in circumcised than genitally intact U.S. males [44]. Additionally, in a U.S. study of males attending STI clinics, circumcised males reported less consistent condom use than genitally intact males [45]. In Denmark, a previous study found that the age at sexual debut, the perceived importance of having a good sex life, partner-related sexual inactivity, and the frequency of sexual activity among the sexually active did not differ between intact and circumcised men. However, a higher proportion of circumcised (38%) than intact (28%) men reported 10 or more sex partners over their lifetime [17]. In the cohort study from Dunedin, New Zealand, circumcised participants had significantly *fewer* sex partners than intact males, and adjustment for this potential confounder did not affect the observed null-association between circumcision status and STI risk in that study [34]. On this background, it is unclear to what extent the present study’s significantly elevated HRs for STIs overall, anogenital warts and syphilis would be explained by more risky sexual behaviors among circumcised men, although higher sociosexual activity and less consistent condom use among circumcised males may have been a contributing factor.

Individual-level socioeconomic variables were unfortunately not available for our analyses. In one of the robustness analyses, we stratified the background rates by municipality-based disposable household income levels to partially overcome this limitation. HRs in this analysis changed only marginally, suggesting that any residual socioeconomic confounding, which was unaccounted for by the municipality-level stratification used, is unlikely to have affected our HRs to any great extent.

In conclusion, our cohort study provides the first nationwide, prospective assessment of the long-term impact on STI risk of non-therapeutic male circumcision carried out in infancy or childhood. Our study provides population-based evidence that early-life circumcision is unlikely to provide protection against HIV or other STIs in males up to the age of 36 years. Rather, in our study circumcision was associated with higher STI rates overall, particularly for anogenital warts and syphilis. Our findings are relevant for ongoing debates on the ethics surrounding genital surgeries on healthy children [46], and should be thoroughly considered among international organizations and stakeholders preparing to expand ongoing adult circumcision programs in Africa in the fight against HIV and other STIs to also cover circumcision of young boys and male infants.

## Data Availability

Due to Danish and EU data protection regulations, original register data must be requested from the respective registers.

## References

[1] WHO. Policy Brief: Preventing HIV through safe voluntary medical male circumcision for adolescent boys and men in generalized epidemics: Recommendations and key considerations. 2020. https://apps.who.int/iris/bitstream/handle/10665/333841/9789240009660-eng.pdf 32986340

[2] Auvert B, Taljaard D, Lagarde E, Sobngwi-Tambekou J, Sitta R, Puren A (2005). Randomized, controlled intervention trial of male circumcision for reduction of HIV infection risk: the ANRS 1265 Trial. PLoS Med.

[3] Bailey RC, Moses S, Parker CB, Agot K, Maclean I, Krieger JN (2007). Male circumcision for HIV prevention in young men in Kisumu, Kenya: a randomised controlled trial. Lancet.

[4] Gray RH, Kigozi G, Serwadda D, Makumbi F, Watya S, Nalugoda F (2007). Male circumcision for HIV prevention in men in Rakai, Uganda: a randomised trial. Lancet.

[5] Weiss HA, Halperin D, Bailey RC, Hayes RJ, Schmid G, Hankins CA (2008). Male circumcision for HIV prevention: from evidence to action?. AIDS.

[6] Tobian AA, Serwadda D, Quinn TC, Kigozi G, Gravitt PE, Laeyendecker O (2009). Male circumcision for the prevention of HSV-2 and HPV infections and syphilis. N Engl J Med.

[7] Gray RH, Serwadda D, Tobian AA, Chen MZ, Makumbi F, Suntoke T (2009). Effects of genital ulcer disease and herpes simplex virus type 2 on the efficacy of male circumcision for HIV prevention: analyses from the Rakai trials. PLoS Med.

[8] Wawer MJ, Tobian AA, Kigozi G, Kong X, Gravitt PE, Serwadda D (2011). Effect of circumcision of HIV-negative men on transmission of human papillomavirus to HIV-negative women: a randomised trial in Rakai. Uganda Lancet.

[9] Loevinsohn G, Kigozi G, Kagaayi J, Wawer MJ, Nalugoda F, Chang LW (2020). Effectiveness of voluntary medical male circumcision for HIV prevention in Rakai, Uganda. Clin Infect Dis.

[10] Morris BJ (2020). Voluntary medical male circumcision proves robust for mitigating heterosexual HIV infection. Clin Infect Dis.

[11] Baeten JM, Donnell D, Ndase P, Mugo NR, Campbell JD, Wangisi J (2012). Antiretroviral prophylaxis for HIV prevention in heterosexual men and women. N Engl J Med.

[12] Mudimu E, Peebles K, Mukandavire Z, Nightingale E, Sharma M, Medley GF (2020). Individual and community-level benefits of PrEP in western Kenya and South Africa: implications for population prioritization of PrEP provision. PLoS ONE.

[13] Saag MS, Gandhi RT, Hoy JF, Landovitz RJ, Thompson MA, Sax PE (2020). Antiretroviral drugs for treatment and prevention of HIV infection in adults: 2020 recommendations of the International Antiviral Society-USA Panel. JAMA.

[14] President’s Emergency Plan For Aids Relief. PEPFAR 2020 Country Operational Plan Guidance for all PEPFAR Countries: PEPFAR. 2019:1–485. https://www.state.gov/wp-content/uploads/2019/11/2019-11-25-COP20-Guidance-Full-Consolidated_Public-2-1.pdf

[15] Hammond T, Carmack A (2017). Long-term adverse outcomes from neonatal circumcision reported in a survey of 1,008 men: an overview of health and human rights implications. Int J Hum Rights.

[16] Kim D, Pang MG (2007). The effect of male circumcision on sexuality. BJU Int.

[17] Frisch M, Lindholm M, Grønbæk M (2011). Male circumcision and sexual function in men and women: a survey-based, cross-sectional study in Denmark. Int J Epidemiol.

[18] Carasco MA, Wilkinson J, Kasdan B, Fleming P (2019). Systematic review of barriers and facilitators to voluntary medical male circumcision in priority countries and programmatic implications for service uptake. Glob Pub Health.

[19] VMMC Experience Project. UN Report - Circumcision campaigns: African opposition and human rights. 2019. https://www.vmmcproject.org/wp-content/uploads/2020/09/VMMC-UN-Report.pdf

[20] Fish M, Shahvisi A, Gwaambuka T, Tangwa GB, Ncayiyana D, Earp BD (2020). A new Tuskegee? Unethical human experimentation and Western neocolonialism in the mass circumcision of African men. Dev World Bioeth.

[21] Rennie S, Gilbertson A, Hallfors D, Luseno WK (2020). Ethics of pursuing targets in public health: the case of voluntary medical male circumcision for HIV-prevention programs in Kenya. J Med Ethics.

[22] Chilimampunga C, Lijenje S, Sherman J, Nindi K, Mavhu W (2017). Acceptability and feasibility of early infant male circumcision for HIV prevention in Malawi. PLoS ONE.

[23] World Health Organization. Manual for early infant male circumcision under local anaesthesia. 2011. ISBN: 978-92-4-150075-3.

[24] Joseph Davey DL, Vermund SH, Wamai R, Phili R, Klausner JD for the early infant male circumcision expert technial group. Why wait? We need to scale up infant male circumcision for global HIV control. AIDS 2016;30(11):1847–8. 10.1097/QAD.0000000000001121 10.1097/QAD.0000000000001121 PMC10767709 27088322

[25] Andale TO, Gachuno O, Awuor TOM (2021). Uptake and outcomes of early infant male circumcision services in four counties in Western Kenya. Afr Health Sci.

[26] Lucas T, Hines JZ, Samuelson J, Hargreave T, Davis SM, Fellows I (2021). Urethrocutaneous fistulas after voluntary medical male circumcision for HIV prevention—15 African Countries, 2015–2019. BMC Urol.

[27] Pedersen CB (2011). The Danish civil registration system. Scand J Public Health.

[28] Frisch M, Simonsen J (2015). Ritual circumcision and risk of autism spectrum disorder in 0- to 9-year-old boys: national cohort study in Denmark. J R Soc Med.

[29] Frisch M, Simonsen J (2018). Cultural background, non-therapeutic circumcision and the risk of meatal stenosis and other urethral stricture disease: two nationwide register-based cohort studies in Denmark 1977–2013. Surgeon.

[30] Lynge E, Sandegaard JL, Rebolj M (2011). The Danish national patient register. Scand J Public Health.

[31] Andersen JS, Olivarius NF, Krasnik A (2011). The Danish national health service register. Scand J Public Health.

[32] Cox DR (1975). Partial likelihood. Biometrika.

[33] Fergusson DM, Boden JM, Horwood LJ (2006). Circumcision status and risk of sexually transmitted infection in young adult males: an analysis of a longitudinal birth cohort. Pediatrics.

[34] Dickson NP, van Roode T, Herbison P, Paul C. Circumcision and risk of sexually transmitted infections in a birth cohort. J Pediatr 2008;152(3):383–7. 10.1016/j.jpeds.2007.07.044 10.1016/j.jpeds.2007.07.044 18280846

[35] American Academy of Pediatrics Task force on circumcision (2012). Circumcision policy statement. Pediatrics.

[36] American Academy of Pediatrics Task force on circumcision (2012). Technical report: male circumcision. Pediatrics.

[37] Frisch M, Aigrain Y, Barauskas V, Bjarnason R, Boddy SA, Czauderna P (2013). Cultural bias in the AAP's 2012 Technical Report and Policy Statement on male circumcision. Pediatrics.

[38] Svoboda JS, Van Howe RS (2013). Out of step: fatal flaws in the latest AAP policy report on neonatal circumcision. J Med Ethics.

[39] Earp BD (2021). Male or female genital cutting: why 'health benefits' are morally irrelevant. J Med Ethics.

[40] Van Howe RS (2013). Sexually transmitted infections and male circumcision: a systematic review and meta-analysis. ISRN Urol.

[41] Epidemiology FL (2000). When an entire country is a cohort. Science.

[42] Frisch M, Moseholm E, Andersson M, Andresen JB, Graugaard C. Sex in Denmark - Key findings from Project SEXUS 2017–2018. Copenhagen, Denmark: Statens Serum Institut and Aalborg University. 2019. ISBN: 978-87-971732-0-6. https://files.projektsexus.dk/2019-10-26_SEXUS-rapport_2017-2018.pdf#page24

[43] Laumann EO, Masi CM, Zuckerman EW (1997). Circumcision in the United States. Prevalence, prophylactic effects, and sexual practice. JAMA.

[44] Miani A, De Bernardo GA, Højgaard AD, Earp BD, Pak PJ, Lanadau AM, Hoppe J, Winterdahl M (2020). Neonatal male circumcision is associated with altered adult socio-affective processing. Helyon.

[45] Crosby R, Charnigo RJ (2013). A comparison of condom use perceptions and behaviours between circumcised and intact men attending sexually transmitted disease clinics in the United States. Int J STD AIDS.

[46] The Brussels Collaboration on Bodily Integrity (2019). Medically unnecessary genital cutting and the rights of the child: moving toward consensus. Am J Bioethics.

